# Preparation of a magnetic graphene/polydopamine nanocomposite for magnetic dispersive solid-phase extraction of benzoylurea insecticides in environmental water samples

**DOI:** 10.1038/s41598-019-45186-z

**Published:** 2019-06-20

**Authors:** Xiaodong Huang, Kexin Qiao, Lingyun Li, Guangyang Liu, Xiaomin Xu, Runhua Lu, Haixiang Gao, Donghui Xu

**Affiliations:** 1 Institute of Vegetables and Flowers, Chinese Academy of Agricultural Sciences, Key Laboratory of Vegetables Quality and Safety Control, Ministry of Agriculture and Rural Affairs of China, Beijing, 100081 China; 2 Department of Applied Chemistry, China Agricultural University, Beijing, 100193 China

**Keywords:** Environmental chemistry, Techniques and instrumentation

## Abstract

A magnetic graphene/polydopamine (MG/PDA) nanocomposite has been prepared and used as sorbent for magnetic dispersive solid-phase extraction (MDSPE) of four benzoylurea insecticides in environmental water samples. The obtained nanocomposites were characterized by transmission electron microscopy, scanning electron microscopy, vibrating sample magnetometry, powder X-ray diffraction, fourier transform infrared spectroscopy, surface area and porosity analysis and thermogravimetric analysis. To investigate the adsorption performance of MG/PDA for target analytes, various parameters affecting the MG/PDA-based MDSPE procedure were optimized. Under the optimal conditions, the established method exhibits good linearity (*R*^2^ ≥ 0.9988) in the concentration range 2.5–500 µg L^−1^. A low limit of detection (0.75 µg L^−1^, signal/noise = 3:1), a low limit of quantification (2.50 µg L^−1^, signal/noise = 10:1), and good precision (intraday relative standard deviation ≤3.6%, interday relative standard deviation ≤4.5%) are also achieved. Finally, the simple, fast, and sensitive sample preparation technique was successfully used to determine benzoylurea insecticides in environmental water samples.

## Introduction

Benzoylurea insecticides (BUIs), as a class of insect growth regulators, have been widely used to control insects because of their capability to interfere with chitin synthesis and inhibit the molting process of target pests^1,2^. Diflubenzuron was the prototype of all BUIs which was firstly discovered in the early 1970s, and 14 more BUIs were prepared and commercialized in the following 40 years of development^3,4^. However, with widespread use and accumulation over time, the residues of BUIs can contaminate water and foods, leading to negative effects on human health, such as carcinogenic and teratogenicity, because of chronic exposure and long-term toxicity^5,6^. Therefore, development of a simple, fast, and sensitive technique to determine BUIs in environmental water is important.

Sample preparation plays a key role in the pesticide residues analysis. To date, a large number of mehods have been researched and developed for the analysis BUIs in water samples, such as dispersive liquid–liquid microextraction, solid-phase extraction (SPE) and solid-phase microextraction (SPME)^7–9^. Dispersive solid-phase extraction (DSPE), which is a miniaturized SPE method introduced in 2003^10^, is considered to be a quick, easy, cheap, effective, robust, and safe sample preparation method. In the original work, the solid sorbent was added to the extractant to eliminate matrix interferences and, consequently, accomplish the purpose of matrix clean-up. More recently, the technique has been applied to directly extract and enrich BUIs^11^. However, the traditional DSPE process is considered to be time consuming and labor intensive because of the essential centrifugation step. An alternative strategy to address this inefficiency in DSPE analysis is to magnetize the sorbent and develop a magnetic DSPE (MDSPE) technique. Recently, several materials have been prepared for the preparing of magnetic sorbents to extract BUIs from different matrixes, such as attapulgite^1^, ionic liquids^12^, and polymers^2,13^.

Graphene, which was discovered in 2004, is a one-atom-thick two-dimensional layer of *sp*^2^-hybridized carbon with a hexagonal packed lattice structure^14^. Owing to the large delocalized π-electron system, graphene can form strong π-stacking interactions with benzene rings, and it is a good candidate as an adsorbent for enrichment of organic molecules with planar structures, such as benzene and five-membered rings^15^. Moreover, Fe_3_O_4_ nanoparticles are used to prepare magnetic graphene (MG) because of their small size, high magnetic susceptibility, and high surface-to-volume ratio^16^. MG is the most intensively studied sorbent material, both as an adsorbent or as a precursor for further modification^17^.

Polydopamine (PDA), as a mimic of adhesive foot protein coming from marine mussels, has attracted tremendous interest as a surface modification reagent to synthesize numerous composites^18^. PDA possesses remarkable chemical and environmental stability, and is usually used to functionalize different materials for adsorption, drug delivery, sensing and catalysis^19^. Furthermore, PDA-functionalized MG has recently been used to enrich phthalates in environmental water samples, indicating that PDA-functionalized MG has potential in many research fields, especially for pesticide enrichment. Therefore, a combination of MG and PDA shows good prospects as an adsorbent for MDSPE of BUIs in water samples.

The aim of this study is to prepare the MG/PDA nanocomposite and develop a simple, fast, and sensitive sample preparation method based on MDSPE coupled with HPLC and diode array detection (DAD) for the determination of BUIs in environmental water samples. The obtained MG/PDA nanocomposite was characterized, and several experimental parameters affecting the MG/PDA-based MDSPE process were investigated and optimized. Finally, the proposed sample preparation method was applied to determinate four BUIs in real water samples.

## Methods

### Reagents and materials

The liquid insecticide standards of the BUIs diflubenzuron, triflumuron, flufenoxuron, and lufenuron (1000 mg L^−1^) were obtained from the Agro-Environmental Protection Institute, Ministry of Agriculture and Rural Affairs of China (Tianjin, China). A standard mixture solution (100 mg L^−1^) of the four benzoylurea insecticides was prepared in acetonitrile, and stored at −20 °C in darkness. HPLC grade methanol, acetonitrile, and ethanol were purchased from Thermo Fisher Scientific (Waltham, Massachusetts, USA). Analytical grade ferric chloride hexahydrate (FeCl_3_·6H_2_O) and ammonium ferrous sulfate hexahydrate [(NH_4_)_2_Fe(SO_4_)_2_·6H_2_O] were purchased from Sinopharm Chemical Reagent Corporation (Shanghai, China). Ammonium hydroxide (NH_3_·H_2_O, mass fraction 25%), sodium chloride (NaCl), and ethanol were acquired from Beijing Chemical Works (Beijing, China). Dopamine hydrochloride and Tris hydrochloride (proteomics grade) were purchased from Beijing Ouhe Technology Corporation (Beijing, China). Graphene was obtained from the Institute of Coal Chemistry, Chinese Academy of Sciences (Taiyuan, China).

### Instrumentation

HPLC analysis was performed with an Agilent 1100 HPLC system equipped with an automatic sample injector and a DAD system (California, USA). A Spursil C18 analytical column (5 mm, 4.6 mm × 250 mm, Dikma Ltd.) was used to separate the insecticides. An acetonitrile–water mixture (75:25, v/v) was prepared as the mobile phase and the flow rate was kept at 1 mL min^−1^ with the column temperature of 30 °C. The DAD wavelength was set to 254 nm. The injection volume was 10 μL.

The obtained materials were characterized by transmission electron microscopy (TEM, JEM-200CX, JEOL, Tokyo, Japan), scanning electron microscopy (SEM, JSM-6300, JEOL, Tokyo, Japan), vibrating sample magnetometry (VSM, Lake Shore 7410, Columbus, USA), powder X-ray diffraction (XRD, D8 Advance, Bruker, Karlsruhe, Germany), fourier transform infrared (FT-IR) spectroscopy (FT-IR-8400, Shimadzu, Kyoto, Japan), thermogravimetric analysis (TGA, STA 449 F3, NETZSCH, Selb, Germany) and surface area and porosity analysis (ASAP2460, Micromeritics, Norcross, USA).

### Preparation of MG/PDA

MG was synthesized by chemical co-precipitation of Fe^2+^ and Fe^3+^ in an alkaline solution in the presence of graphene. First, graphene (0.6 g) was dispersed to 80 mL of FeCl_3_·6H_2_O (1.764 g) solution after a 1 h ultra-sonication. Subsequently, 0.646 g of (NH_4_)_2_Fe(SO_4_)_2_·6H_2_O was dissolved in the suspension, and kept mechanical stirring (300 rpm) for 30 min under the protection of N_2_ at 90 °C. Then, 2.4 mL of NH_3_·H_2_O solution was added into the mixture drop by drop followed by another 30 min mechanical stirring. Finally, the black product was isolated using a neodymium magnet and alternating washed three times with ultrapure water and ethanol, respectively. The MG was dried at 60 °C for 24 h in a vacuum oven.

The MG/PDA nanocomposite was fabricated as follows. First, 0.4 g of MG was suspended in 210 mL of ethanol/ultrapure water (4:3, v/v) under ultrasonication for 10 min. Subsequently, dopamine hydrochloride (1.6 g) was dissolved in the suspension, followed by mechanical stirring (800 rpm) at room temperature for 30 min. Then, Tris buffer solution (60 mL, pH 8.5, 25 mM) was added to the mixture, and then continuously stirring for 10 h to perform the polymerization reaction under room temperature. Finally, the mixed solution was separated under the external magnetic field, and kept alternative washing by ultrapure water and ethanol for three times. After that, the obtained MG/PDA nanocomposites were dried at 60 °C for 24 h in a vacuum oven.

### MDSPE procedure

**Figure 1:**
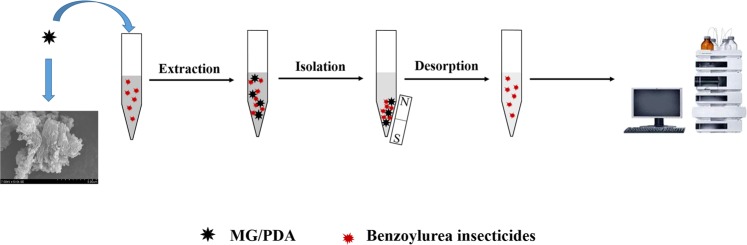
Schematic diagram of the MDSPE procedure with the MG/PDA composite.

The workflow of the complete MDSPE process is presented in Fig. 1. Typically, MG/PDA (10 mg) was added to a 10 mL centrifuge tube which contains 8 mL of spiked water sample, followed by 0.5 min of vortex to accomplish the extraction process. After that, the magnetic sorbents were collected by a neodymium magnet which was placed on the outside bottom of the tube, then the clear supernatant was discarded. Subsequently, acetonitrile (1.5 mL) was added into the tube and kept vortexing for 30 s to elute BUIs from the adsorbent. After the MG/PDA nanocomposites were retrieved, the desorption solvent was then transferred to another new centrifuge tube. Finally, the desorbed solution was evaporated to dryness under a gentle stream of N_2_ at 40 °C. The residues were redissolved in 100 μL of acetonitrile, and 10 μL of the redissolved solution was injected into the HPLC–DAD system for analysis.

### Sample preparation

Real environmental water samples were obtained from three different reaches of the Tiantang River (Beijing China): the upper reach of the Tiantang River on the Nangezhuang Enterprise Park (16:20, 30 January 2016,), the lower reach of Tiantang River on the Nangezhuang Enterprise Park (13:37, 30 January 2016), and the Nangezhuang Electroplating Factory reach (15:04, 30 January 2016). All of these real water samples were filtered through a 0.45 µm polytetrafluoroethylene membrane filter and stored in darkness under 4 °C condition.

## Results and Discussion

### Characterization of MG/PDA

**Figure 2:**
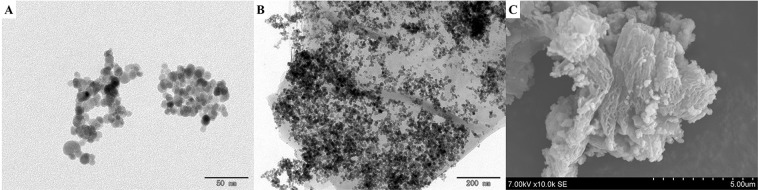
TEM images of (**A**) Fe_3_O_4_ and (**B**) MG. (**C**) SEM image of MG/PDA.

TEM and SEM were used to investigate the Fe_3_O_4_, MG, and MG/PDA morphologies. TEM images of Fe_3_O_4_ and MG and a SEM image of MG/PGA are shown in Fig. 2. The Fe_3_O_4_ nanoparticles are appeared as slight aggregation because of superparamagnetism, and densely deposited on the surface of MG (Fig. 2B) with an average diameter of nearly 10 nm (Fig. 2A). Moreover, the prepared MG/PDA nanocomposite exhibit a rough morphology on surface due to the fold of graphene layer and the surface modification of PDA (Fig. 2C), suggesting that the synthetic magnetic nanocomposite material possesses good prospect of applicability in the adsorption of BUIs.

**Figure 3:**
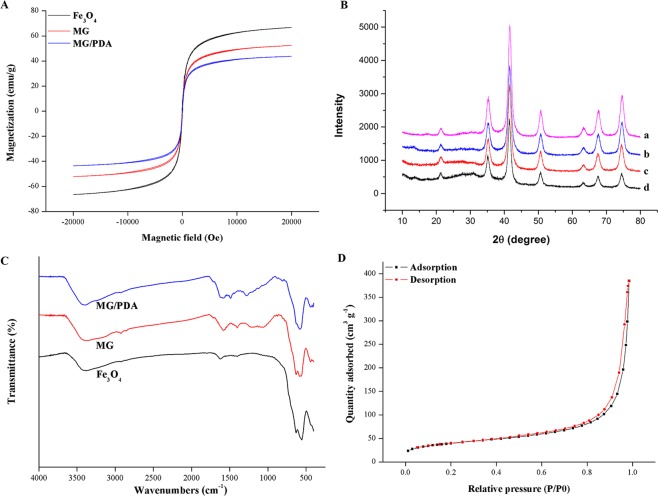
(**A**) Magnetic curves of the prepared materials. (**B**) XRD patterns of (a) Fe_3_O_4_, (b) MG, (c) MG/PDA and (d) MG/PDA treated with the developed MDSPE method. (**C**) FT-IR spectra of the prepared materials. (**D**) N_2_ adsorption–desorption isotherms of MG/PDA.

The magnetic behavior of the obtained magnetic nanocomposite was studied by VSM. As can be seen from the magnetic hysteresis loops of Fe_3_O_4_, MG, and MG/PDA in Fig. 3A, the coercivity and remanence values are both zero, indicating that the prepared materials have typical magnetic properties and easily to be collected under the magnetic field. Furthermore, the saturation magnetization values of Fe_3_O_4_, MG, and MG/PDA, are 66.7, 52.3, and 43.7 emu g^−1^, respectively. The XRD patterns of the synthetic magnetic materials are shown in Fig. 3B. The diffraction pattern of MG/PDA is in accordance with the patterns of the other component materials (Fe_3_O_4_, MG). This indicates good retention of Fe_3_O_4_ nanoparticles in MG/PDA after polymerization. The FT-IR spectra of the obtained magnetic nanocomposites are presented in Fig. 3C. The adsorption band at 584 cm^−1^ in all the spectra can be attributed to the Fe–O vibration, suggesting that the MG/PDA nanocomposites were well contained Fe_3_O_4_ nanoparticles^20^. In MG/PDA spectrum, the adsorption band at 1582, 1489, and 1281 cm^−1^ corresponding to the stretching vibrations of C=C, N–H, and C–O of PDA, respectively^21^.

**Figure 4:**
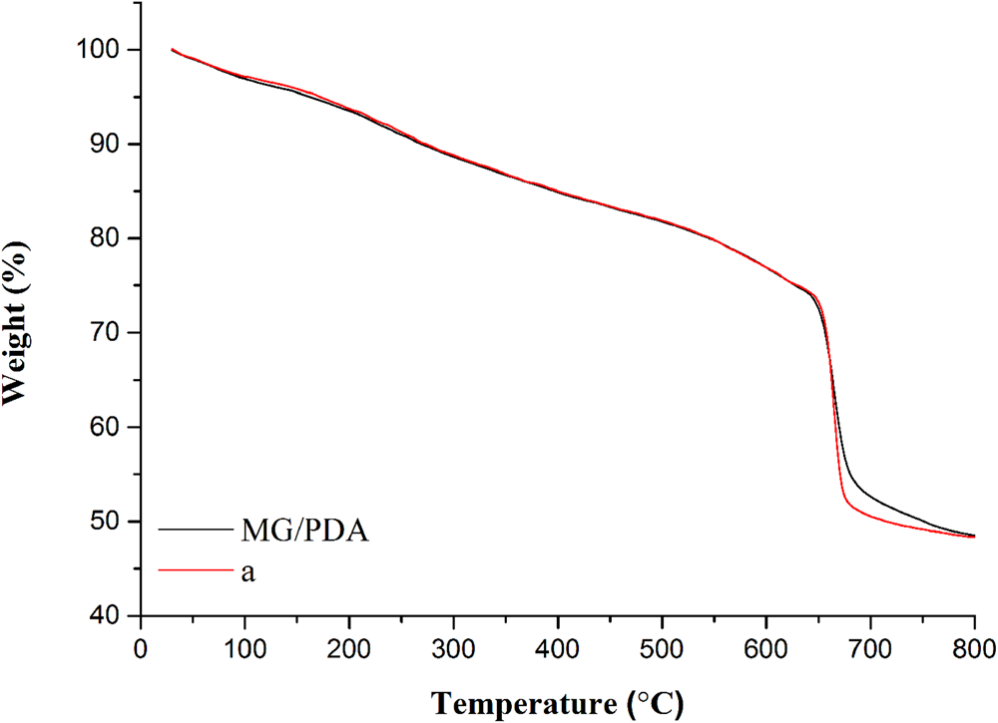
TGA curves of MG/PDA and (a) treated with the developed MDSPE method.

The pore properties of the MG/PDA nanocomposite were researched by N_2_ adsorption–desorption, and the N_2_ adsorption–desorption isotherms are shown in Fig. 3D. The coexistence of different pores ranging from meso- to macropores can be attributed to N_2_ adsorption slowly increasing at low relative pressures (*P*/*P*_0_ < 0.8) and sharply increasing at high relative pressures (0.8 < *P*/*P*_0_ < 1.0)^22^. The pore volume and BET surface area of MG/PDA are 0.595 mL g^−1^ and 137.77 m^2^ g^−1^, respectively. Furthermore, the thermostability of the MG/PDA was confirmed by TGA from room temperature to 800 °C. As shown in Fig. 4, the thermogram of MG/PDA shows two decomposition stages. The former stage could be assigned to water evaporation from room temperature to 120 °C. The latter stage was corresponded to the degradation of PDA in the range of 650–680 °C, which indicates the successful coated of PDA on the surface of MG^23^.All of the VSM, XRD, FT-IR, N_2_ adsorption–desorption characterization and TGA results suggest that the MG/PDA nanocomposite was successfully synthesized and possesses large surface area and large total pore volume.

### Optimization of the MDSPE parameters

The as-synthesized MG/PDA nanocomposite was used as an adsorbent for MDSPE of BUIs from aqueous samples. Several experimental parameters were optimized to achieve satisfactory extraction of the BUIs from water samples, including the sorbent amount, extraction time, salt effect, and desorption conditions. All of the experiments were performed in triplicate.

**Figure 5:**
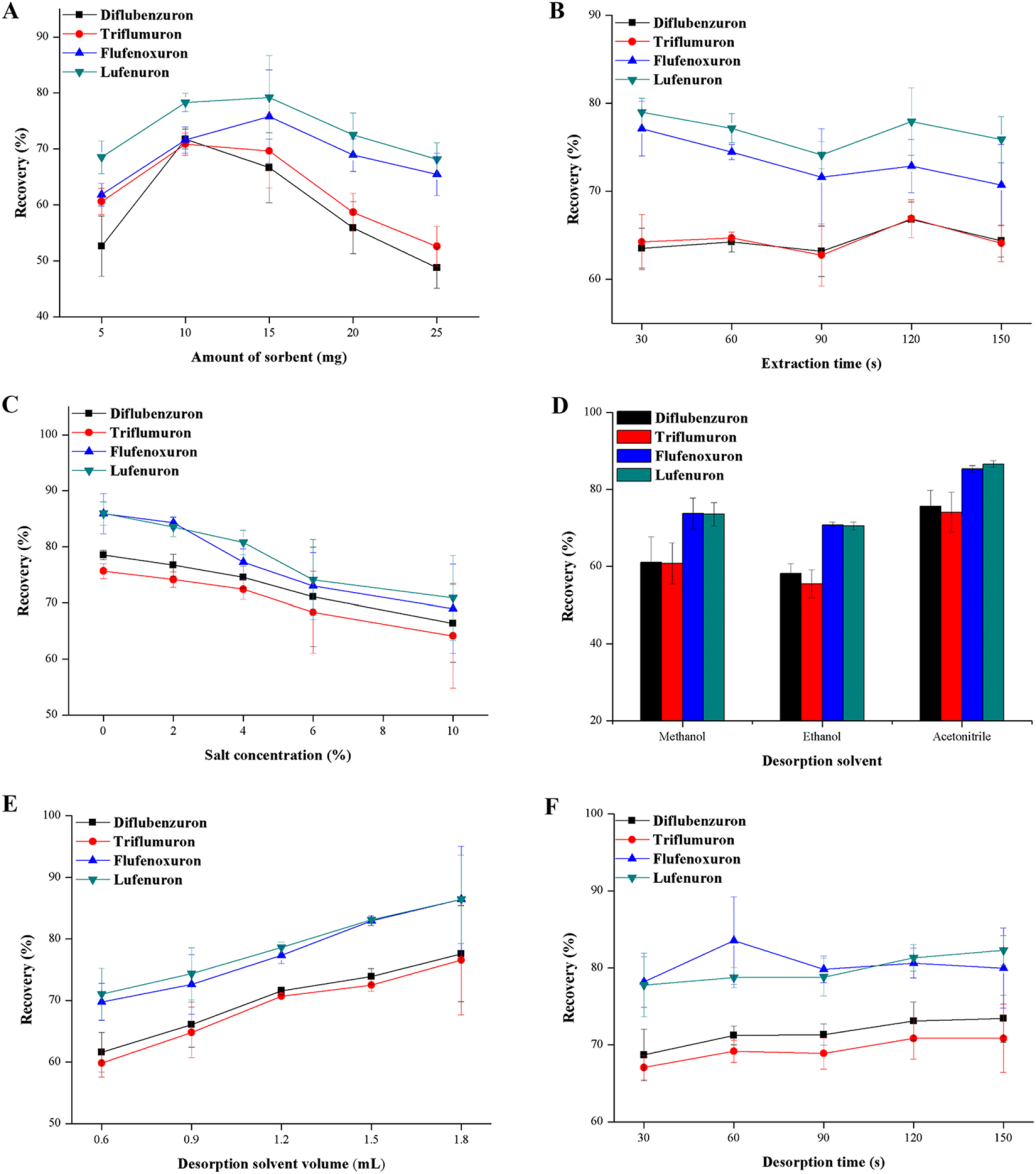
Effect of different parameters on the extraction efficiency of BUIs from water: (**A**) amount of adsorbent, (**B**) extraction time, (**C**) salt concentration, (**D**) desorption solvent, (**E**) desorption solvent volume, and (**F**) desorption time.

In general, the adsorbent amount significantly affects the extraction efficiency. An insufficient amount of the adsorbent results in unsatisfactory extraction efficiency of target analytes from sample solutions. Conversely, excessive adsorbent has no promotion effect on the extraction efficiency, and might require more desorption solvent to elute the analytes^24^. In the present work, the dosage of adsorbent on the extraction efficiency was studied by adding different amounts of the sorbent ranging from 5 to 25 mg. The results are shown in Fig. 5A. The extraction recoveries of all of BUIs increase with increasing dosage of adsorbent from 5 to 10 mg, but the recoveries slightly decrease with a further increase in the sorbent dosage owing to incomplete desorption. Therefore, 10 mg is the optimal amount of the adsorbent.

Owing to the probable affection on the adsorption equilibrium between the sample solution and the sorbent, extraction time always plays to be a kernel factor in the MDSPE procedure. In this study, five different vortex time in the range 30–150 s were investigated after dispersing MG/PDA in the sample solution by vortex agitation at 3000 rpm. The effect of the extraction time on the MDSPE is shown in Fig. 5B. The results show that 30 s is sufficient and no more promotion on the extraction efficiency was observed by further increasing the vortex time. Hence, 30 s is the optimal adsorption time.

Generally, a solution with a suitable ionic strength can decrease the solubility of analytes in the aqueous phase and therefore enhance the binding affinity between the analytes and the sorbent^25^. In this study, the ionic strength of the water sample was adjusted by adding several content of NaCl (0–10%, w/v). The effect of the salt concentration on the MDSPE is shown in Fig. 5C. The extraction recoveries of all of the analytes slightly decrease with increasing addition of NaCl. As to the analysis above, no NaCl is needed for the MG/PDA-based MDSPE procedure.

Desorption is a crucial step for MDSPE to elute the target analytes from the sorbent. To achieve satisfactory extraction efficiency, parameters affecting the elution performance of MDSPE for BUIs were investigated, such as the selection of desorption solvent, volume of desorption solvent, and desorption time.

To study the effect of eluent on the MG/PDA-based MDSPE process, acetonitrile, methanol and ethanol were investigated as potential desorption solvent. The extraction efficiencies of the three desorption solvents are presented in Fig. 5D. Acetonitrile exhibits the best promotion effect on the extraction efficiency, and therefore was chosen as the optimum eluent for the MDSPE process.

To optimized the consumption of eluent for the desorption procedure, 0.6 to 1.8 mL of acetonitrile was used to study the elution effect on the extraction efficiency. As illustrated on Fig. 5E, the extraction efficiencies on four analytes show an increasing trend with gradually increasing volume of the desorption solvent. However, the fortified recoveries of the analytes for 1.5 mL desorption solvent volume exceed 70% and show more satisfying parallelism compared with 1.8 mL. Therefore, the consumption of the eluent was set to 1.5 mL.

To investigate the influence of the desorption time on the MG/PDA-based MDSPE efficiency, experiments were conducted with vortex times of 30, 60, 90, 120, and 150 s. As can be seen in Fig. 5F, the extraction efficiencies do not significantly change with increasing vortex time. In consideration of the operational efficiency, 30 s was selected as the optimum desorption time for the MDSPE process.

### Reusability of the adsorbent

**Figure 6:**
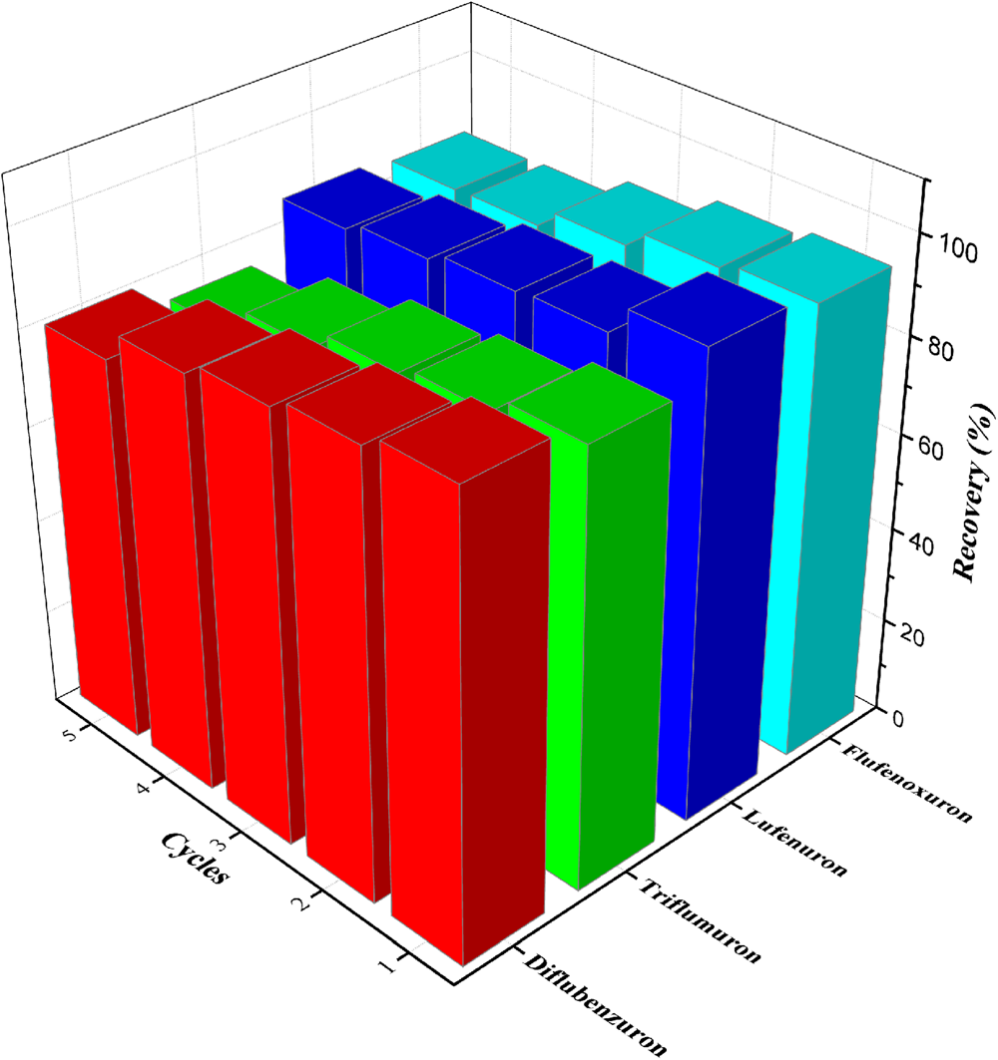
Regeneration and reusability of MG/PDA.

In order to evaluate the reusability of the MG/PDA, the recycling experiments were performed under the optimal experimental conditions. After the complete adsorption-desorption, 1.5 mL of acetonitrile was used to wash the adsorbent by vortexing 30 s before the next cycle of the developed MDSPE procedure. As can be seen from Fig. 3B (d), the XRD pattern of the MG/PDA nanocomposite shows no significant difference before and after one cycle of adsorption-desorption experiment, which indicated that the crystalline structure remains unchanged. Moreover, the Fig. 4 shows that the TGA curves of MG/PDA exhibited no significant difference before or treated with one cycle of the developed MDSPE technique. As shown in Fig. 6, the extraction efficiency remained satisfactory after five recycles, and all of these results suggested that the adsorbent possesses good stability and reusability.

### Method evaluation

To evaluate the performance of the developed MDSPE–HPLC–DAD method for analysis of BUIs, the analytical characteristics, such as the linear ranges, LODs, LOQs, and RSDs, were determined under the optimized experimental conditions. A series of spiked water samples with BUI concentrations in the range 2.5–500 μg L^−1^ were prepared in triplicate (*n* = 3) to construct the working curves and calculate other analytical parameters. As shown in Table 1, the proposed method exhibits good linearity with coefficients of determination (*R*^2^) ranging from 0.9988 to 0.9999. The LODs of the BUIs calculated at a signal-to-noise (*S*/*N*) ratio of 3 achieved by gradually decreasing the spiked concentrations are all 0.75 µg L^−1^. The LOQs determined from the minimum concentration of the linearity at *S*/*N* = 10 are all 2.50 µg L^−1^. To confirm the precision of the suggested technique, six replicate blank water samples spiked with 10 µg L^−1^ of each of the BUIs were analyzed. The results indicate that the fortified recoveries of the BUIs range from 75.7% to 86.0%, and the intraday and interday precision range from 0.9% to 3.6% and 3.4% to 4.5%, respectively. Comprehensive analysis of the above results, we can see that the established MDSPE–HPLC–DAD technique exhibit high sensitivity and good repeatability.

**Table 1:** Analytical parameters of MG/PDA as an adsorbent for MDSPE of four BUIs in ultrapure water samples.

Compounds	Linear equation	Linearity (µg L^−1^)	*R* ^2^	LOD (µg L^−1^)	LOQ (µg L^−1^)	Recovery (%)	RSD (%) (n = 6)	
							Intraday	Interday
Diflubenzuron	Y = 1.6629X + 1.4268	2.5–500	0.9999	0.75	2.50	78.5	0.9	3.6
Triflumuron	Y = 1.4320X + 2.9768	2.5–500	0.9999	0.75	2.50	75.7	1.3	4.5
Flufenoxuron	Y = 1.0060X + 2.0595	2.5–500	0.9988	0.75	2.50	85.9	3.6	3.4
Lufenuron	Y = 1.3989X + 6.0964	2.5–500	0.9992	0.75	2.50	86.0	2.1	3.6

### Comparison of the MG/PDA-based MDSPE method with other reported methods

To evaluate the potential of the MG/PDA nanocomposite as an adsorbent for MDSPE of BUIs, the proposed method was compared with some recently published methods coupled with HPLC for determination of BUIs in environmental water samples (Table 2). The proposed method using MG/PDA as the adsorbent needs the shortest extraction time and shows the best precision.

**Table 2:** Comparison of different methods for analysis of BUIs.

Method	Extraction sorbent	Sorbent amount	Extraction time	Recovery (%)	RSD (%)	LODs (μg L^−1^)	Ref.
SPE-HPLC	TiO_2_ nanotube	—	45 min	82–100	—	0.062–0.21	^8^
SPME-HPLC	β-CDP@ Fe_3_O_4_	16 mg	25 min	87.3–112.5	1.5–5.3	0.02–0.05	^13^
SPME-HPLC	MMF/MAED fiber	—	70 min	70.9–118	1.5–9.8	0.026–0.075	^9^
MLLE/DSPE-LC	Fe_3_O_4_/SiO_2_/ILs	3 mg	4 min	73.2–85.8	2.2–4.5	0.67–1.46	^26^
MDSPE-HPLC	MG/PDA	10 mg	30 s	70.6–91.6	0.3–5.9	0.75	This work

### Real sample analysis

**Figure 7:**
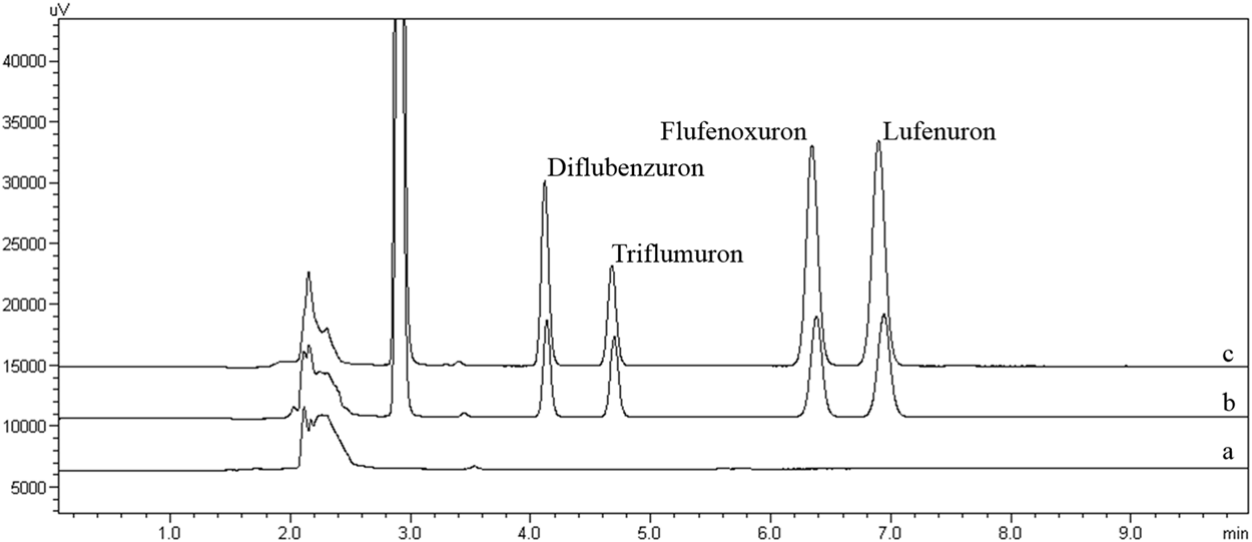
Typical chromatograms of blank (**a**) and spiked water (**b**) 100 μg L^−1^; (**c**) 250 μg L^−1^) samples from the upper reach of the Tiantang River on the Nangezhuang Enterprise Park.

To evaluate the applicability of the developed MG/PDA-based method, several environmental water samples obtained from three different reaches of the Tiantang River were selected as real water samples for BUI determination. The analytical results and the recoveries of the target BUIs are given in Table 3. The fortified recoveries of the four BUIs are 70.6–91.6% with precision (RSDs) values of 0.3–5.9%. Typical chromatograms of the blank and spiked water samples from the upper reach of Tiantang River in the Nangezhuang Enterprise Park are shown in Fig. 7.

**Table 3:** Analytical results of determination of BUIs in real water samples.

Analytes	Spiked concentration (µg L^−1^, n = 3)	Reach 1^a^		Reach 2^b^		Reach 3^c^	
		Recovery (%)	RSD (%)	Recovery (%)	RSD (%)	Recovery (%)	RSD (%)
Diflubenzuron	0	ND^d)^	—	ND	—	ND	—
	100	72.7	3.8	73.7	2.2	71.8	5.9
	250	70.7	3.3	70.6	5.1	75.1	2.1
Triflumuron	0	ND	—	ND	—	ND	—
	100	77.0	0.3	79.1	3.7	75.8	3.4
	250	74.1	1.8	73.1	4.9	77.1	1.0
Flufenoxuron	0	ND	—	ND	—	ND	—
	100	83.4	1.4	84.4	1.7	79.2	0.4
	250	80.8	2.5	75.0	5.0	77.2	3.3
Lufenuron	0	ND	—	ND	—	ND	—
	100	88.1	2.3	91.1	1.4	91.6	5.0
	250	91.2	4.9	86.1	3.0	90.8	2.9

^a^Upper reach of the Tiantang River on the Nangezhuang Enterprise Park.

^b^Lower reach of the Tiantang River on the Nangezhuang Enterprise Park.

^c^Nangezhuang Electroplating Factory reach of the Tiantang River.

^d^ND means not detected.

## Conclusions

The MG/PDA nanocomposite has been successfully synthesized and then used as sorbent for MDSPE of BUIs in environmental water samples. The characterization results show that the as-synthesized MG/PDA nanocomposite possesses high superparamagnetism, large BET surface area, and large total pore volume, which mean that it has prospect in the rapid separation of BUIs from water samples. Combined with HPLC–DAD, the as-developed simple, fast, and sensitive sample preparation technique shows acceptable extraction efficiencies, good linearity, low detection and quantification limits, good accuracy and precision for extraction of BUIs in water samples. This study will support the research of other types of advanced PDA-functionalized magnetic materials for the efficient adsorption of organic contaminants from complex matrix solution samples.
